# A BI-NATIONAL COMPARISON OF FEDERAL SAFETY NET PROGRAMS FOR LOW-INCOME ELDERLY IN THE U.S. AND MEXICO

**DOI:** 10.1093/geroni/igz038.251

**Published:** 2019-11-08

**Authors:** Emma Aguila, Jaqueline L Angel

**Affiliations:** 1 University of Southern California, Los Angeles, California, United States; 2 University of Texas at Austin, Austin, Texas, United States

## Abstract

Population aging in Mexico as in the United States is expected to accelerate over the next thirty years, and the proportion of individuals 65 and older will triple to approximately 20 percent by 2050 in both nations. Older people of Mexican origin are at high risk of protracted periods of poor health, a reality exacerbated by poverty. We use the Health and Retirement Study (HRS 2012-2014, N= 2,575) and Mexican Health and Aging Study (MHAS 2012-2015; N=16,131) to compare profiles of older Mexican-origin recipients of income supplements. We find Mexican immigrants are lower-income, less healthy, and less likely to receive supplements than Mexican origin in U.S. In contrast, return migrants are more likely to receive supplements than non-migrants in Mexico. Income supplement recipients are more likely to receive Medicaid and Seguro Popular. We discuss implications of financing safety net programs and the potential dependency burden in two countries aging rapidly.

